# The phenotypic changes of γδ T cells in COVID‐19 patients

**DOI:** 10.1111/jcmm.15620

**Published:** 2020-08-30

**Authors:** Lei Lei, Hongbo Qian, Xiaofang Yang, Xingzhe Zhang, Dan Zhang, Tongxin Dai, Rui Guo, Lin Shi, Yanbin Cheng, Baojun Zhang, Xiaobo Zhou, Jinsong Hu, Yaling Guo

**Affiliations:** ^1^ Department of Pathogenic Microbiology and Immunology School of Basic Medical Sciences Xi'an Jiaotong University Health Science Center Xi'an China; ^2^ Department of Clinical Laboratory The 8th hospital of Xi'an Xi'an China; ^3^ Key Laboratory of Environment and Genes Related to Diseases Xi’an Jiaotong University Health Science Center Xi'an China; ^4^ Institute of Infection and Immunity Translational Medicine Institute Xi’an Jiaotong University Health Science Center Xi’an China; ^5^ Department of Cell Biology and Genetics Xi'an Jiaotong University Health Science Center Xi'an China

**Keywords:** activation, COVID‐19, innate immunity, SARS‐CoV‐2, γδ T cells

## Abstract

A novel pneumonia‐associated respiratory syndrome named coronavirus disease‐2019 (COVID‐19), which was caused by SARS‐CoV‐2，broke out in Wuhan, China, in the end of 2019. Unfortunately, there is no specific antiviral agent or vaccine available to treat SARS‐CoV‐2 infections. The information regarding the immunological characteristics in COVID‐19 patients remains limited. Here, we collected the blood samples from 18 healthy donors (HD) and 38 COVID‐19 patients to analyze changes on γδ T cell population. In comparison with HD, the γδ T cell percentage decreased, while the activation marker CD25 expression increased in response to SARS‐CoV‐2 infection. Interestingly, the CD4 expression was upregulated in γδ T cells reflecting the occurrence of a specific effector cell population, which may serve as a biomarker for the assessment of SARS‐CoV‐2 infection.

## 1 INTRODUCTION

A severe pneumonia‐associated respiratory syndrome spread rapidly in Wuhan, China, at the end of 2019. A novel coronavirus, officially named severe acute respiratory syndrome coronavirus 2 (SARS‐CoV‐2), was identified as the cause of emerging cases of severe pneumonia.^1^, ^2^, ^3^ Officially named by WHO, the coronavirus infection disease‐19 (COVID‐19) outbreak was listed as a public health emergency of international concern. The virus has so far caused 81 896 confirmed cases and 3287 deaths in China according to WHO by 8 April 2020. COVID‐19 has rapidly spread in more than 180 countries worldwide, including Italy, Iran, Japan and the United States.

SARS‐CoV‐2 is an enveloped positive‐sense RNA virus, which belongs to the family of coronaviruses including SARS‐CoV and MERS‐CoV.^4^, ^5^ Currently, there is no specific antiviral agent or vaccine available to treat SARS‐CoV‐2 infections. Clinical treatments for COVID‐19 patients are primarily supportive and symptomatic treatments. There are several existing antiviral agents that can be repurposed to develop effective interventions against this novel coronavirus.^6^ However, toxicology studies and clinical trials are required for potential uses in the clinic. According to the pathological reports for COVID‐19, SARS‐CoV‐2 mainly caused inflammatory responses in the lungs.^7^ Several studies showed that COVID‐19 patients developed lymphopenia and rising pro‐inflammatory cytokines in severe cases.^8^, ^9^ Inflammation can be triggered when innate and adaptive immune cells detect SARS‐CoV‐2 infection. Innate T cells can provide a first line of defence against pathogens. However, how innate T cells respond to SARS‐COV‐2 infection remains unclear.

Among innate immune cells, γδ T cells proliferate rapidly and respond to pathogens by inducing apoptosis, mediating antigen presentation and immune regulation.^10^ In healthy adult humans, γδ T cells represent 1%‐10% of total circulating lymphocytes, predominately displaying the CD4 and CD8 double‐negative phenotype.^11^ However, in some cases, a fraction of γδ T cells can express either CD4 or CD8.^12^, ^13^, ^14^ The γδ T cells in Itk‐ and Id3‐deficient mice exhibited an increase in CD4 and CD44 expression, as well as cytokine production (IL‐4, IFNγ or IL‐17), indicating an enhanced effector function in the context of infection or disease occurrence.^15^, ^16^ γδ T cells do not recognize classical peptide antigens, their TCRs are non‐MHC restricted, and they can respond to pathogen‐associated molecular patterns and produce cytokines in the absence of TCR ligands.^17^ Furthermore, γδ T cells can defend against viral infection by secreting IFNγ and upregulating the expression of NKG2D, perforin, granzyme B and FasL, etc^18^, ^19^

In many infections, the number of γδ T cells increases both locally and systemically a few days post‐infection. A study found that the ratio of γδ T cells among total lymphocytes in the lungs significantly increased in mice infected with influenza A (H1N1) virus 3 days following infection.^19^ This observation suggests that γδ T cells play an important role in the host immune response. During acute HIV infection, previous studies showed that the expression of the activation marker, CD25, is significantly increased on γδ T cells,^14^ whereas various viruses may have different effects on the activation pattern of γδ T cells.^20^, ^21^

**Figure 1:**
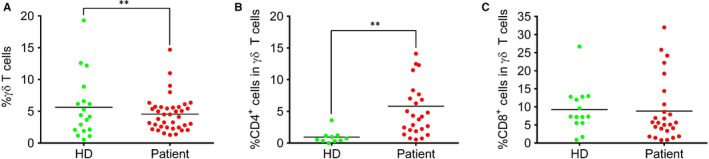
The percentage of γδ T cell populations in the blood of healthy donors and COVID‐19 patients (A)The percentage of total γδ T cells; (B) the percentage of CD4 + γδ T cells; (C) the percentage of CD8 + γδ T cells

To demonstrate how γδ T cells behave upon SARS‐CoV‐2 infection, we analysed the peripheral blood mononuclear cells (PBMC) samples from 38 patients and focused on the characterization of γδ T cell phenotypes. We showed that upon infection, the percentage of γδ T cells in the lymphocyte from peripheral blood mononuclear cell (PBMC) isolated from COVID‐19 patients was drastically decreased when compared with healthy donors (Figure 1A). Although the percentage of γδ T cells typically increases during the acute or early stages of other viral infections, we observed a decrease of γδ T cells in symptomatic patients. This may be due to the fact that various types of viruses impact γδ T cells in different ways. Therefore, it is likely that γδ T cell response, including proliferation and cellularity, is dependent on the specific types of viral infections. It is also possible that most patients in this study showed mild symptom such as fever, as opposed to serious illnesses featuring pneumonia.

Since CD4 γδ T cells are linked to the effector phenotype, we evaluated the proportion of both CD4 and CD8 γδ T cells. Interestingly, we found that in comparison with healthy donor (HD) group, the percentage of CD4 γδ T cells within the γδ T cell population increased dramatically, while CD8 γδ T remained unchanged in COVID‐19 patients (Figure 1B,C). The increase of CD4 γδ T cells indicates that in response to SARS‐CoV‐2 infection, this particular subset of γδ T cells may play a role in antigen presentation and facilitate the activation of adaptive immune cells, which has been demonstrated in different models.^22^ The data also suggest that this subset of γδ T cells can immediately respond to viral infection, providing the first line of defence as shown in macrophages and dendritic cells. Therefore, γδ T cells may act as a bridge between innate and adaptive immunity in response to SARS‐CoV‐2 infection.^23^

**Figure 2:**
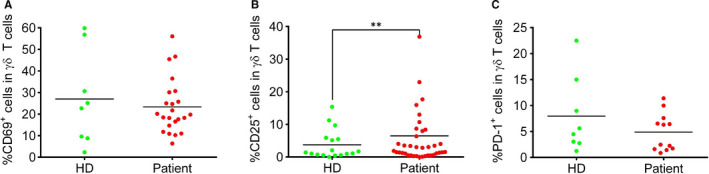
The expression of activation markers in γδ T cells from the blood of healthy donors and COVID‐19 patients (A) The percentage of CD69 + γδ T cells; (B) the percentage of CD25 + γδ T cells; (C) the percentage of PD‐1 + γδ T cells

In COVID patients, we further observed that γδ T cells exhibited a strong activation phenotype in COVID‐19 patients based on CD25 expression (Figure 2B). However, the early activation marker CD69 showed no difference between the patients and HD group (Figure 2A). It is possible CD69 is expressed strongly earlier during infection, followed by reversion to the quiescent state during prolonged recovery. Since we observed a decreased percentage of γδ T cells, we suspect that γδ T cells developed an exhausted phenotype. However, the expression of PD‐1 did not differ in γδ T cells between HD and COVID‐19 patients (Figure 2C).

In summary, γδ T cells are able to immediately respond to SARS‐CoV‐2 infection and upregulate the activation marker CD25. γδ T cells may act in parallel to other innate cells to mediate both direct and indirect defences against SARS‐CoV‐2. In addition, the increased expression of CD4 in γδ T cells may serve as a biomarker for the assessment of SARS‐CoV‐2 infection.

## 2 MATERIALS AND METHODS

### 2.1 Ethics statement

This study was approved by the Research Ethics Commission of the Eighth Hospital of Xi'an (20190730‐1346). All subjects signed informed consent forms upon admission to the hospital. In this study, all cases were taken from the Eighth Hospital of Xi'an (Xi'an, Shaanxi Province, People's Republic of China).

### 2.2 Patients

The study included 18 healthy controls and 40 patients from February 18 to March 4. In the HD group, the median age is 39.06 ± 4.26 years, with equal numbers of male and female subjects. The median age of the patient group was 45.08 ± 4.06 years, including 23 male patients (60.53%) and 15 female patients (39.47%). The 38 patients enrolled were all confirmed to have SARS‐CoV‐2 infection using PCR tests on throat swab specimens. All patients were categorized as mild by clinical manifestations.

### 2.3 Flow cytometry analysis

The antibodies (Abs) used in the flow cytometry analysis are as follows: FITC anti‐human TCR γ/δ (B1), APC/Cyanine7 anti‐human CD4 (OKT4), PerCP/Cyanine5.5 anti‐human CD8 (SK1), APC anti‐human CD25 (BC96), PE anti‐human CD69 (FN50) and APC anti‐human CD279 (PD‐1) (EH12.2H7) were purchased from BioLegend. Blood cells were stained with Abs in the dark at room temperature for 15 minutes and analysed on a FACSCanto II flow cytometer (BD Biosciences). FlowJo 8 (company information? Treestar?) was used for data analysis.

### 2.4 Statistical analysis

Student's *t* test was performed for two group analysis using GraphPad Prism 7.0 software. * and ** stands for *P* < .05 and *P* < .01, respectively.

## CONFLICT OF INTERESTS

We declare no competing interests.

## AUTHOR CONTRIBUTION

**Lei Lei:** Writing‐original draft (equal). **Hongbo Qian:** Data curation (equal). **Xiaofeng Yang:** Writing‐original draft (supporting). **Xingzhe Zhang:** Methodology (supporting). **Dan Zhang:** Software (supporting). **Tongxin Dai:** Data curation (supporting). **Rui Guo:** Data curation (supporting). **Lin Shi:** Writing‐review & editing (supporting). **Yanbin Cheng:** Writing‐review & editing (supporting). **Baojun Zhang:** Funding acquisition (equal); Project administration (equal); Writing‐review & editing (equal). **Xiaobo Zhou:** Project administration (equal). **Jinsong Hu:** Data curation (equal); Project administration (equal). **Yaling Guo:** Project administration (equal); Writing‐review & editing (equal).

## Data Availability

The data sets used and analysed in the current study are available from the corresponding author upon reasonable request.
